# Porous, Ventricular Extracellular Matrix-Derived Foams as a Platform for Cardiac Cell Culture

**DOI:** 10.1089/biores.2015.0030

**Published:** 2015-10-01

**Authors:** Valerio Russo, Ehsan Omidi, Abbas Samani, Andrew Hamilton, Lauren E. Flynn

**Affiliations:** ^1^ Department of Chemical Engineering, Queen's University, Kingston, Ontario, Canada.; ^2^ Human Mobility Research Centre, Kingston General Hospital, Kingston, Ontario, Canada.; ^3^ Biomedical Engineering Graduate Program, The University of Western Ontario, London, Ontario, Canada.; ^4^ Department of Electrical and Computer Engineering, The University of Western Ontario, London, Ontario, Canada.; ^5^ Department of Surgery, Kingston General Hospital, Kingston, Ontario, Canada.; ^6^ Department of Chemical and Biochemical Engineering, The University of Western Ontario, London, Ontario, Canada.; ^7^ Department of Anatomy and Cell Biology, Schulich School of Medicine & Dentistry, The University of Western Ontario, London, Ontario, Canada.

**Keywords:** biomaterials, cell culture, extracellular matrix, stem cells, tissue engineering

## Abstract

To more closely mimic the native cellular microenvironment, 3D scaffolds derived from the extracellular matrix (ECM) are being developed as alternatives to conventional 2D culture systems. In the present study, we established methods to fabricate nonchemically cross-linked 3D porous foams derived entirely from decellularized porcine left ventricle (DLV) for use as an *in vitro* cardiac cell culture platform. Furthermore, we explored the effects of physically preprocessing the DLV through mechanical mincing versus cryomilling, as well as varying the ECM concentration on the structure, composition, and physical properties of the foams. Our results indicate that the less highly processed minced foams had a more cohesive and complex network of ECM components, enhanced mechanical properties, and improved stability under simulated culturing conditions. To validate the DLV foams, a proof-of-concept study was conducted to explore the early cardiomyogenic differentiation of pericardial fat adipose-derived stem/stromal cells (pfASCs) on the minced DLV foams relative to purified collagen I gel controls. Differentiation was induced using a modified cardiomyogenic medium (MCM) or through stimulation with 5-azacytidine (5-aza), and cardiomyocyte marker expression was characterized by immunohistochemistry and real-time reverse transcriptase-polymerase chain reaction. Our results indicate that early markers of cardiomyogenic differentiation were significantly enhanced on the DLV foams cultured in MCM, suggesting a synergistic effect of the cardiac ECM-derived scaffolds and the culture medium on the induction of pfASC differentiation. Furthermore, in analyzing the response in the noninduced control groups, the foams were observed to provide a mildly inductive microenvironment for pfASC cardiomyogenesis, supporting the rationale for using tissue-specific ECM as a substrate for cardiac cell culture applications.

## Introduction

Mammalian cell culture studies for cardiac applications have conventionally been performed in 2D on tissue culture polystyrene (TCPS) coated with fibronectin,^1^ laminin,^2,3^ or fetal bovine serum (FBS).^4^ However, these simplified culture models do not closely replicate the complex 3D microenvironment of the native extracellular matrix (ECM), which plays a critical role in directing *in vivo* cell behavior.^5^ In studies with primary cardiomyocytes, culturing on TCPS can cause changes in cell phenotype and electrophysiological characteristics that can impact the fidelity of these model systems.^6,7^ Furthermore, while there is growing interest in the cardiomyogenic differentiation of stem cells, standard 2D culture methods yield highly mixed cell populations, including nonfunctional cardiomyocytes displaying markers of an immature phenotype and residual undifferentiated cells.^8–10^

Recognizing the critical importance of the ECM in mediating cell function, there is a need to develop culture systems that better mimic the native cellular microenvironment. In this context, decellularization techniques can provide a means of obtaining tissue-specific ECM for culture studies.^11^ There is compelling evidence to support that cell viability, proliferation, and/or lineage-specific differentiation can be enhanced when cells are cultured on ECM-derived bioscaffolds.^12–14^ However, using decellularized tissues as *in vitro* cell culture substrates can have limitations, including the naturally heterogeneous and dense structure of the ECM.^15^ To design more highly tunable ECM-derived platforms, our group and others have been developing alternative scaffold formats, such as porous foams and hydrogels.^14,16–18^ Depending on the processing methods applied, these scaffolds can retain tissue-specific ECM components while providing a more uniform cell interface and enabling greater control over physical properties.

In the current study, our primary objective was to design and characterize novel 3D porous foams derived from decellularized porcine left ventricle (DLV). In contrast to previous approaches relying on pepsin digestion to synthesize cardiac ECM-derived gels^18–20^ and foams,^15^ we utilized α-amylase digestion, which enables the isolation of highly polymerized collagen from tissues.^21,22^ Furthermore, we compared the effects of coarse mincing versus cryomilling the lyophilized DLV before enzymatic digestion on the structure, composition, and physical properties of the foams synthesized over a concentration range of 20–50 mg/mL.

Following physical characterization, we conducted proof-of-concept studies to test the DLV foam as an *in vitro* cell culture platform by comparing the early cardiomyogenic differentiation of human pericardial fat adipose-derived stem/stromal cells (pfASCs) on the DLV foams and type I collagen gels. There is increasing interest in the use of ASCs for cardiovascular regeneration due to their relative abundance and accessibility,^23^ and we selected the pfASC population as recent evidence suggests that ASCs sourced from this depot may have enhanced cardiomyogenic potential.^24^ Differentiation was induced through stimulation with a modified cardiomyogenic medium (MCM)^10^ or the epigenetic modifier, 5-azacytidine (5-aza),^10,25^ and the cellular response was characterized over 14 days by immunohistochemical (IHC) staining for early cardiac protein markers as well as real-time reverse transcriptase-polymerase chain reaction (RT-PCR) analysis of cardiomyogenic gene expression.

## Materials and Methods

### Materials

Unless otherwise stated, all chemicals were purchased from Sigma-Aldrich Canada (Oakville, Canada) and used as received.

### Porcine ventricular myocardium decellularization

Fresh porcine hearts were obtained from Quinn's Meats Ltd. (Yarker, Canada). Decellularization was performed using methods adapted from Wainwright et al.^26^ Briefly, the left ventricles from a total of 10 porcine hearts were excised and the fibrous outermost layers of the epicardium and endocardium were carefully removed to maximize tissue exposure to the decellularization reagents. The samples were then pooled and subjected to four cycles of freeze–thaw in hypotonic freezing buffer solution (pH 8.0) containing 10 mM Tris base and 5 mM ethylenediaminetetraacetic acid (EDTA). Next, the tissue was cut into 4–5-mm^3^ pieces and treated with two cycles of 6 h in 0.25% trypsin-EDTA (Life Technologies, Burlington, Canada), 20 h in 4% sodium deoxycholate, and 20 h in 3% Triton X-100 under orbital shaking at 37°C, with repeated rinsing in phosphate-buffered saline (PBS) between solution changes. All solutions were supplemented with 1% antibiotic–antimycotic solution (Life Technologies) and 0.01 mM phenylmethanesulfonyl fluoride (PMSF). The DLV was rinsed extensively in distilled water and lyophilized using a ModulyoD freeze dryer (Thermo Scientific, Waltham, MA).

### DLV suspension preparation and foam fabrication

**FIG. 1.**
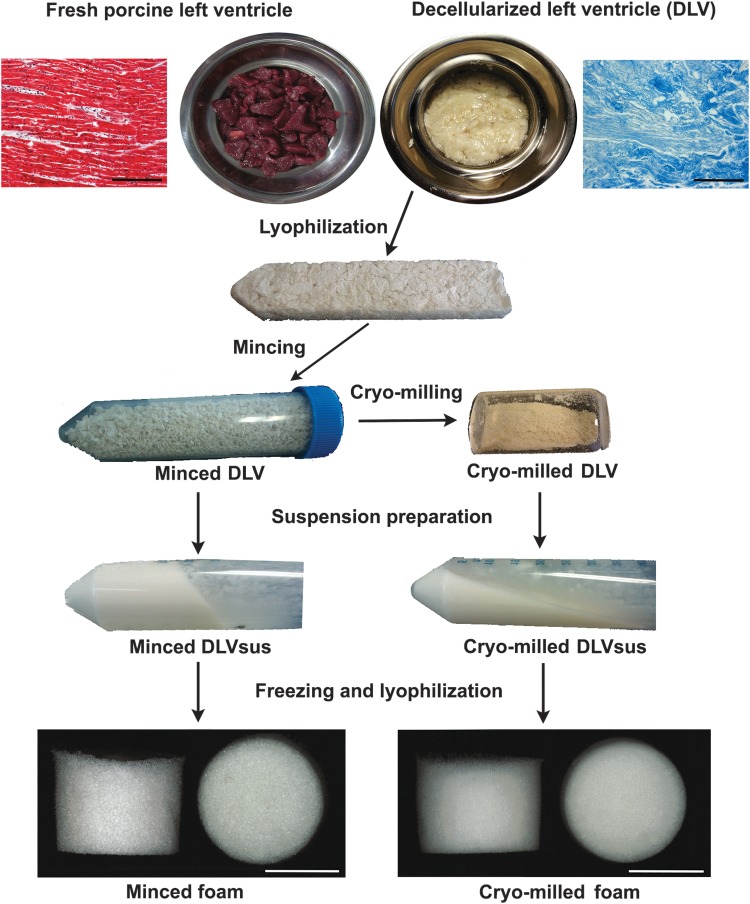
Overview of the foam fabrication methods. Top: Masson's trichrome staining demonstrated that a collagen-rich matrix was obtained following decellularization of the porcine ventricular myocardium (scale bars represent 200 μm). The lyophilized decellularized porcine left ventricle (DLV) was further processed through mechanical mincing or cryomilling and used to create homogeneous DLV suspensions (DLVsus) through α-amylase digestion and homogenization. Bottom: porous minced and cryomilled DLV foams were generated from the DLVsus by freezing and lyophilization (scale bars represent 5 mm).

DLV foams were fabricated using methods adapted from Yu et al.^14^ An overview of the fabrication process is shown in Figure 1. Briefly, DLV suspensions (DLVsus) were prepared using two processing methods. In the first approach, the lyophilized DLV was cut into ∼2-mm^3^ fragments with scissors to obtain minced DLV. In the second method, the minced DLV was further processed through cryomilling for 3 min at 2550 rpm with a Mikro-Dismembrator S ball mill (Sartorius, Göttingen, Germany) to yield cryomilled DLV. A 50 mg/mL stock DLVsus of minced or cryomilled DLV was prepared by digesting the processed DLV in 1% (w/w) α-amylase from *Aspergillus oryzae* in 0.22 M NaH_2_PO_4_ buffer under continuous agitation for 3 days at room temperature. The solid phase was then homogenized in 0.22 M acetic acid with a PowerGen Model 125 homogenizer (Fisher Scientific, Ottawa, Canada). Varying concentrations of DLVsus were prepared by diluting the 50 mg/mL stock with sterile 0.2 M acetic acid. For the physical characterization studies, minced and cryomilled DLV foams were fabricated at concentrations of 20, 30, 40, and 50 mg DLV per milliliter within 48-well plates using 500 μL of DLVsus. For the mechanical testing studies, larger foams were fabricated within 24-well plates using 1.2 mL of DLVsus. For the cell culture studies, 30 mg/mL minced DLV foams were fabricated in 48-well plates using 300 μL of DLVsus. The suspension was frozen within the molds at −20°C overnight and lyophilized for 24 h. For all assays, *n* indicates the number of technical replicates per trial and *N* indicates the number of trial repeats.

### Scanning electron microscopy

Scanning electron microscopy (SEM) was used to characterize the internal structure of freeze-fractured DLV foams (*n* = 2, *N* = 2). The dried samples were mounted on aluminum studs and pulse coated with gold for 20 min before imaging using a JEOL JSM-840 microscope (JEOL, Inc., Peabody, MA) at an accelerating voltage of 10 kV and a working distance of 15 mm.

### Histological and IHC analyses

Minced and cryomilled DLV foams fabricated with 50 mg/mL DLVsus were fixed in 4% paraformaldehyde overnight before paraffin embedding and sectioning (5 μm thickness). DLV and native porcine myocardium were included as controls. Masson's trichrome staining was performed to assess decellularization and ECM structure using a Zeiss Invertoskop 40C optical microscope (*n* = 2, *N* = 2). The collagen structure in the DLV foams was further characterized by Picrosirius red staining using established methods.^27^ The sections were analyzed by polarized light microscopy using a Nikon OPTIPHOT-POL microscope (Nikon, Tokyo, Japan) and imaged with an Infinity 2–3 CCD camera (Lumenera, Ottawa, Canada) at 10× magnification.

The ECM composition was assessed through IHC costaining for laminin/collagen I and collagen IV/fibronectin, following the experimental design in Table 1 (*n* = 2, *N* = 2). Briefly, the sections were deparaffinized, subjected to antigen retrieval, and blocked with 10% goat serum for 1 h at room temperature before incubation in the primary antibodies overnight at 4°C. No primary antibody controls were included for all samples. Detection was performed by incubating the samples in the secondary antibodies for 1 h at room temperature in the dark. The samples were mounted in fluoroshield mounting medium with DAPI (Abcam, Toronto, Canada) and images were acquired using a Zeiss Imager M1 microscope with an AxioCam MRm (Carl Zeiss Microscopy, Jena, Germany) at 20× magnification.

**Table 1.** **Experimental Design for Scaffold Immunostaining**

Staining	Antigen retrieval	Primary antibody/dilution	Secondary antibody/dilution
LN/Coll I	Enzymatic (0.05% Trypsin/EDTA)	Polyclonal rabbit anti-LN/1:75 (Cat. No. ab11575)	Alexa Fluor^®^ 488 goat anti-rabbit/1:150 (Cat. No. A-11008)
		Monoclonal mouse anti-Coll I/1:500 (Cat. No. ab90395)	Alexa Fluor^®^ 555 goat anti-mouse/1:500 (Cat. No. A-21422)
Coll IV/FN	Heat mediated (Dako buffer)	Polyclonal rabbit anti-Coll IV/1:100 (Cat. No. ab6586)	Alexa Fluor 488 goat anti-rabbit/1:200 (Cat. No. A-11008)
		Monoclonal mouse anti-FN/1:500 (Cat. No. ab6328)	Alexa Fluor 555 goat anti-mouse/1:500 (Cat. No. A-21422)

All primary antibodies were purchased from Abcam (Toronto, Canada) and secondary antibodies were purchased from Life Technologies (Burlington, Canada).

LN, laminin; Coll I, collagen I; Coll IV, collagen IV; FN, fibronectin.

### Assessment of physical properties

#### Equilibrium water content

The equilibrium water content (EWC) of the DLV foams was determined using established protocols (*n* = 4, *N* = 5).^14^ To prepare the samples, the lyophilized DLV foams were weighed and subjected to a controlled rehydration process. Briefly, the foams were incubated in 100% ethanol within a vacuum chamber overnight and then transferred into a 50% ethanol solution and incubated for an additional 24 h. The foams were then rinsed repeatedly and incubated overnight in fresh distilled water before recording the hydrated mass.

#### Swelling ratio

To assess swelling, the DLV foams were imaged in both the lyophilized and rehydrated states with a Zeiss Discovery V12 stereomicroscope (Carl Zeiss Microscopy) (*n* = 4, *N* = 2). The dimensions of the foams were determined from the stereomicroscope images using ImageJ analysis software to determine the dry (*V*_D_) and hydrated (*V*_H_) volumes of the cylindrical foams. The swelling ratio (SR) was then calculated according to the following formula:

\begin{align*}{ \rm SR} = ( V_{ \rm H} / V_{ \rm D} ) \times 100 \%\end{align*}

#### Porosity

DLV foam porosity was measured following published protocols using a modified Archimedes method employing isopropanol as the solvent (*n* = 4, *N* = 2).^28^

#### Mechanical testing

The Young's modulus of the minced and cryomilled DLV foams (*n* = 3) was measured in hydrated samples using an unconstrained indentation method with a plane-ended indenter, as described previously.^14^ In brief, the indenter was connected to a linear LAL-30 servo motor (SMAC, Carlsbad, CA) driven by a 6K2 motor controller device (Parker Hannifin Corporation, Rohnert Park, CA). The forces resulting from sample indentation were measured with a high-precision LCL-113 model load cell (Omega, Quebec, Canada) with a capacity of 113 g. The Young's modulus was calculated taking into account the initial linear portion of the force–displacement curve using the following formula:

\begin{align*}E = \kappa S\end{align*}

where *κ* is a conversion factor calculated using an inverse finite element (FE) modeling algorithm and *S* is the measured force–displacement slope.^29^ The FE mesh was created using the actual dimensions of the foams.

#### In vitro stability assay

Protein release from the minced and cryomilled DLV foams was quantified by analyzing the supernatant at 72 h, 7, 14, 21, and 28 days during incubation in Ringer's simulated physiological fluid (8.6 mg/mL NaCl, 0.3 mg/mL KCl, and 0.33 mg/mL CaCl_2_ in deionized water) at 37°C using a Lowry's Protein Assay Kit (Thermo Scientific) (*n* = 4, *N* = 2). Percentage mass remaining at 28 days was also determined based on the dry mass of the foams.

### Human pfASC isolation and culture

Pericardial adipose tissue samples were collected from patients undergoing cardiac surgery at the Kingston General Hospital in Kingston, Canada, with approval from the Research Ethics Board at Queen's University (REB# CHEM-002-07). Human pfASCs were isolated using published methods.^30^ The primary isolates were plated on tissue culture flasks (Corning^®^ 75 cm^2^; Fisher Scientific, Oakville, Canada) at 30,000 cells/cm^2^ in Dulbecco's modified Eagle's medium (DMEM):Ham's F12 medium supplemented with 10% FBS (HyClone; Fisher Scientific) and 100 U/mL penicillin and 0.1 mg/mL streptomycin (1% pen-strep; Life Technologies). The cells were expanded in culture (37°C, 5% CO_2_) with medium changes every 2–3 days. Passage 4 (P4) pfASCs were used for all studies. Before scaffold seeding, the pfASC immunophenotype was confirmed by single marker staining for CD44, CD90, CD105, CD73, CD34, CD31, CD146, and CD45 using a Guava easyCyte 8HT flow cytometer (Millipore, Etobicoke, Canada) as previously described (Supplementary Fig. S1).^23^

### Scaffold seeding and culture

Minced foams fabricated with a DLVsus concentration of 30 mg/mL were used for the culture studies. As a control, collagen I gels were fabricated with PureCol^®^ purified bovine collagen type I (Advanced BioMatrix, San Diego, CA), following the manufacturer's instructions. All scaffolds were equilibrated overnight in complete low-glucose DMEM (DMEM-lg, 1 g/L glucose) supplemented with 10% FBS and 1% pen-strep. For scaffold seeding, the pfASCs were trypsin released and counted using the Guava^®^ ViaCount assay (Millipore, Billerica, MA). Each DLV foam and collagen I gel was seeded with 5 × 10^5^ pfASCs in 150 μL of complete DMEM-lg and incubated for 72 h to promote cell attachment. As an additional control group for the IHC studies, 4 × 10^5^ pfASCs were seeded on gelatin-coated glass cover-slips (18 × 18 mm; VWR, Radnor, PA). After 72 h, cardiomyogenic differentiation was induced in one set of the DLV foams, collagen I gel controls, and gelatin-coated cover-slips by culturing for 7 and 14 days in MCM comprising DMEM-lg supplemented with 10% FBS, 1% pen-strep, 1.0 mg/mL human insulin (Cat. No. A11382II; Life Technologies), 0.55 mg/mL transferrin, 0.5 μg/mL sodium selenite, 50 mg/mL bovine serum albumin, 0.47 μg/mL linoleic acid, 10^−4^ M ascorbate, and 10^−9^ M dexamethasone.^10^ Another complete set of samples was induced by exposure to a 24-h pulse of 10 μM 5-aza in DMEM-lg supplemented with 10% FBS and 1% pen-strep, followed by continued culturing in complete DMEM-lg for 7 or 14 days. A third set of samples was maintained in complete DMEM-lg for the duration of the study as a noninduced control. For all groups, the medium was changed every 2 days.

### IHC analysis of cardiomyogenic protein expression

At 7 and 14 days postinduction, the DLV foams and collagen I gels were fixed overnight in 4% paraformaldehyde before paraffin embedding and sectioning (*n* = 2, *N* = 2). Glass cover-slips were fixed in 4% paraformaldehyde for 1 h and stored in PBS at 4°C until use (*n* = 3, *N* = 2). For antigen retrieval, the cover-slips were incubated for 15 min at 95°C in a Tris-urea (TU) buffer at pH 9.5 containing 100 mM Tris and 5% w/v urea. Following deparaffinization, antigen retrieval, and blocking, all samples were costained overnight at 4°C with anti-Nkx2.5 and anti-ventricular myosin light chain 2 (Mlc2v) (Table 2). Human atrial appendage was included as a tissue positive control (Supplementary Fig. S2). The samples were mounted in DAPI mounting medium and imaged using a Zeiss Imager M1 microscope with an AxioCam MRm (Carl Zeiss Microscopy) at 60× magnification no later than 1 day poststaining.

**Table 2.** **Experimental Design for Pericardial Fat Adipose-Derived Stem/Stromal Cell Immunostaining**

Staining	Antigen retrieval	Primary antibody/dilution	Secondary antibody/dilution
Nkx2.5/Mlc2v	Heat-mediated 3D (TE buffer pH 9)	Polyclonal rabbit anti-Nkx2.5/1:500 (Cat. No. ab35842)	Alexa Fluor 488 goat anti-rabbit/1:500 (Cat. No. A-11008)
	2D (TU buffer pH 9.5)	Monoclonal mouse anti-Mlc2v/1:100 (Cat. No. ab89594)	Alexa Fluor 555 goat anti-mouse/1:200 (Cat. No. A-21422)

All primary antibodies were purchased from Abcam and secondary antibodies were purchased from Life Technologies.

TE, Tris-EDTA; TU, Tris-urea.

### Quantitative RT-PCR of cardiomyogenic gene expression

Unless otherwise stated, all reagents were purchased from Life Technologies. Quantitative analysis of the expression of cardiac troponin T type II (*cTnT*, Cat. No. Hs00943911_m1), myosin heavy chain 6 (*Mhc6*, Cat. No. Hs01101425_m1), and atrial natriuretic peptide (*Anp*, Cat. No. Hs00383230_g1), along with the housekeeping genes β*-actin* (Cat. No. Hs01060665_g1) and tata box-binding protein (*TBP*, Cat. No. Hs00427620_m1), was performed using TaqMan^®^ real-time RT-PCR technology. Gene expression was assessed at 7 and 14 days postinduction using a StepOnePlus™ Real-Time PCR System in triplicate induced and noninduced DLV foam and collagen I gel samples (*n* = 3).

Total RNA was extracted from the scaffolds and reverse transcribed using published methods.^31^ A TaqMan Fast Advanced Master Mix (Cat. No. 4444557) was used for the quantitative polymerase chain reaction (qPCR) experiments following the manufacturer's instructions. Samples from each time point were analyzed in triplicate on the same MicroAmp^®^ Fast Optical 96-well reaction plate (Cat. No. 4346906). Blank and no reverse transcriptase (no-RT) samples were included as negative controls. Expression of each gene of interest was normalized to the geometric mean of the expression of β-*actin* and *TBP* using the comparative C_t_ method with the collagen I gel group at day 7 (*cTnT*) or 14 (*Anp* and *Mhc6*) as the calibrator.^32^

### Statistical analyses

Statistical analyses were carried out using GraphPad Prism version 6 (San Diego, CA). All data are expressed as the mean ± standard deviation and differences were considered significant at *p* < 0.05. Statistical analyses were performed by one- or two-way ANOVA with a Tukey's *post hoc* test.

## Results

### Characteristics of DLVsus

Masson's trichrome staining of the DLV confirmed decellularization yielded a collagen-rich matrix (Fig. 1). Over the concentration range studied, the minced DLV produced a more viscous DLVsus than the cryomilled DLVsus (Fig. 1).

### Foam structure and composition

**FIG. 2.**
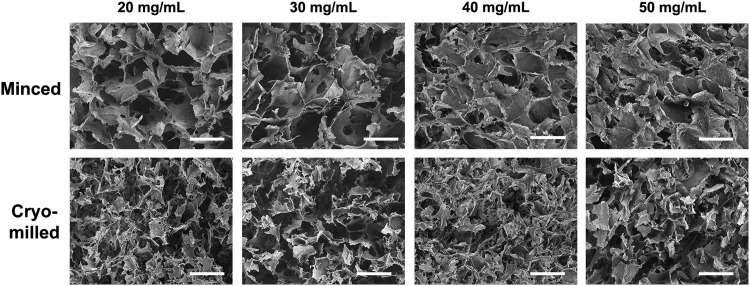
Representative scanning electron microscopy images of the minced and cryomilled DLV foams demonstrating a more intact network in the minced foams and qualitatively enhanced porosity at lower concentrations. Scale bars represent 200 μm.

**FIG. 3.**
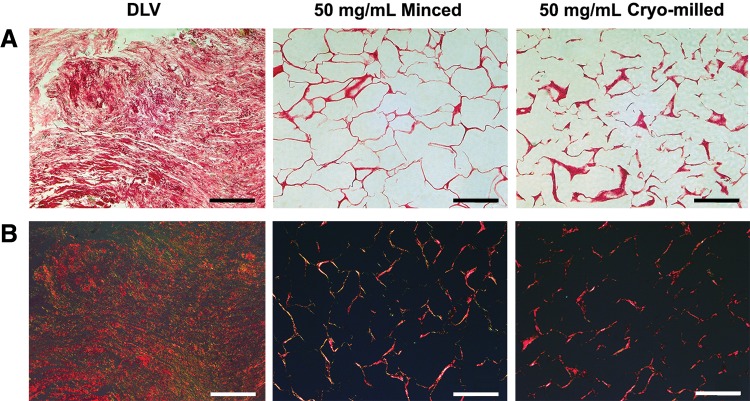
Representative Picrosirius red staining of the intact DLV as well as minced and cryomilled DLV foams under **(A)** bright-field and **(B)** polarized light microscopy. The minced foams had a more cohesive network with a well-defined pore structure as well as a more heterogeneous collagen composition, including both red and green/yellow fibers when visualized under polarized light. Scale bars represent 200 μm.

Both fabrication methods yielded porous foams with a geometry defined by the freezing mold (Fig. 1). SEM analysis revealed that the cryomilled foams contained a more fragmented network compared with the minced foams, which was particularly evident at the lower concentrations that were qualitatively more porous (Fig. 2). The structural differences between the groups were corroborated by the Picrosirius red staining, which showed a more continuous open porous network in the minced foams (Fig. 3). Furthermore, under polarized light microscopy, the minced foams were shown to include both red and yellow/green fibers, suggesting a more heterogeneous composition/organization of thin and thick fibers compared with the cryomilled foams where only red fibers were observed (Fig. 3).^33^

**FIG. 4.**
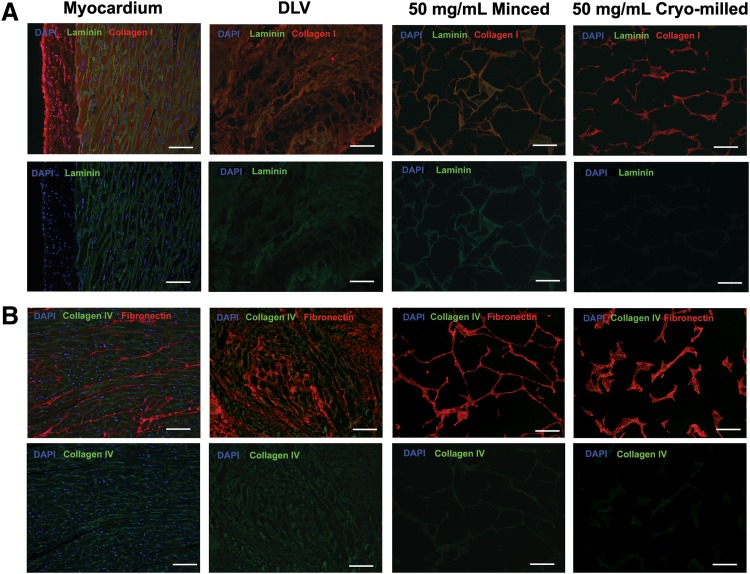
Representative immunohistochemical (IHC) costaining for **(A)** laminin (green)/collagen I (red) and **(B)** collagen IV (green)/fibronectin (red) with DAPI counterstaining (blue) in the native porcine myocardium, intact DLV, and minced and cryomilled DLV foams. Both types of DLV foams incorporated collagen I and fibronectin. Collagen IV was conserved in the intact DLV, but was not detected in either type of foam. Laminin was detected in the intact DLV and minced foams, but not the cryomilled foams. No nuclei were observed through DAPI staining of the intact DLV or DLV foams. Scale bars represent 100 μm.

In terms of ECM composition, collagen I was the predominant component of the DLV foams (Fig. 4A). In addition, both types of foams stained strongly positive for fibronectin (Fig. 4B). While collagen IV was detected in the intact myocardium and DLV, it was not visualized in the foams, suggesting that this component may have been degraded during foam fabrication. Interestingly, positive staining for laminin was detected in the myocardium, DLV, and minced foams, but not the cryomilled foams (Fig. 4A).

### Physical characteristics of the foams

**FIG. 5.**
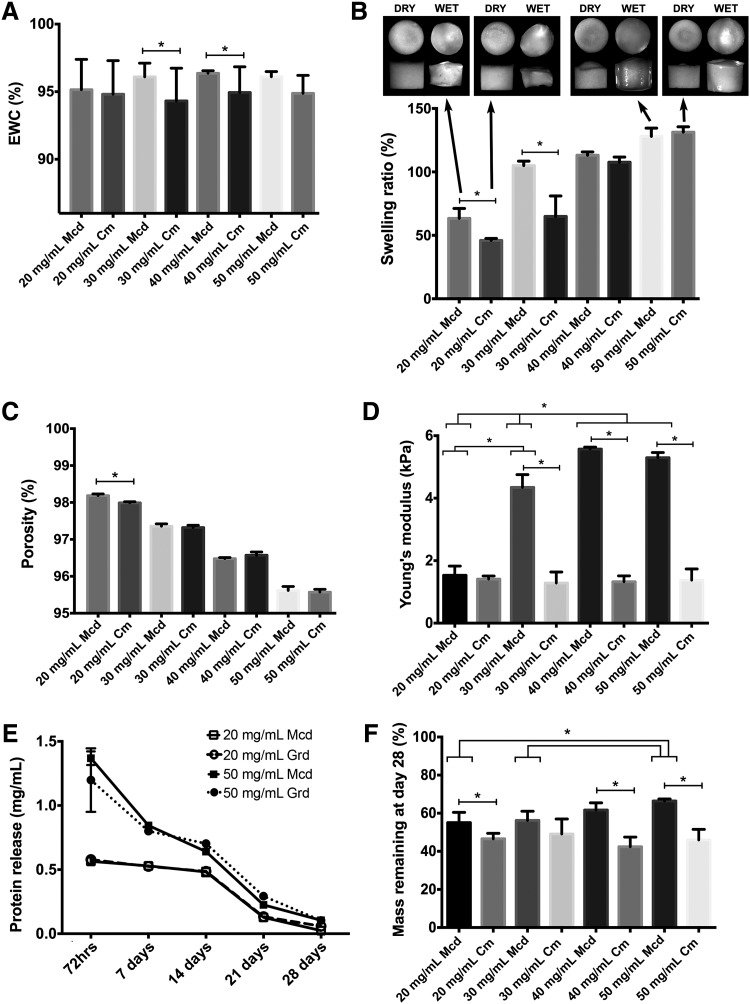
Physical characterization data for the minced (Mcd) and cryomilled (Cm) foams. All data are expressed as mean ± standard deviation (SD). * Significantly different (*p* < 0.05). **(A)** Equilibrium water content (*n* = 4, *N* = 5). **(B)** Swelling ratio and representative macroscopic images of 20 and 50 mg/mL foams before and after rehydration. In assessing the effect of concentration, all differences were statistically significant for both types of foams with the exception of the 30 and 40 mg/mL minced foams and the 40 and 50 mg/mL minced foams (*n* = 4, *N* = 2). **(C)** Porosity. All differences as a result of DLVsus concentration were statistically significant for both types of foams (*n* = 4, *N* = 2). **(D)** Young's modulus of the hydrated foams. For the cryomilled foams, there were no statistically significant differences as a result of DLVsus concentration (*n* = 3). **(E)** Representative trends in protein release during the stability study shown for the 20 and 50 mg/mL foam groups as measured by the Lowry's protein assay of the supernatant (*n* = 4, *N* = 2). **(F)** Percentage mass remaining at day 28 in the culture stability study. For the cryomilled foams, there were no statistically significant differences as a result of DLVsus concentration (*n* = 4, *N* = 2).

In the physical characterization studies, the EWC of the foams was generally in the range of 95% for all of the conditions tested (Fig. 5A). In terms of swelling, both types of foams fabricated at concentrations of 40 and 50 mg/mL swelled slightly in distilled water while macroscopically maintaining their original geometry (Fig. 5B). At the 30 mg/mL concentration, the minced foams demonstrated minimal changes in shape or size, while the cryomilled foams showed significant shrinkage. At the lowest concentration of 20 mg/mL, both types of foams reduced in volume following rehydration, although the minced foam group was more resistant to contraction.

The porosity of both types of foams was generally high, with a lowest value of 95.4% ±0.1% for the 50 mg/mL cryomilled foam group (Fig. 5C). While the porosity was not significantly affected by the processing methods, an inverse relationship was observed between foam porosity and DLV concentration. Despite the similar porosity between the two groups, the Young's moduli of the minced foams were statistically higher than the cryomilled foams for all concentrations except 20 mg/mL (Fig. 5D). More specifically, the modulus of the minced foams ranged from 1.53 ± 0.29 to 5.57 ± 0.06 kPa, whereas the modulus of the cryomilled foams was not dependent on the DLVsus concentration and never exceeded 1.5 kPa.

In assessing foam stability of the foams, protein loss over 28 days followed a similar trend for both types of scaffolds, with higher protein levels measured in the supernatant at the earlier time points for all groups (Fig. 5E). However, the percentage mass remaining at 28 days was generally higher in the minced foams, suggesting enhanced stability in this group (Fig. 5F).

### ASC cardiac protein expression on DLV foams

To validate the DLV foams as a culture platform for cardiac applications, initial studies focused on comparing early cardiac protein marker expression in human pfASCs cultured on the DLV foams and purified collagen I gel controls. The minced foams were selected for these studies based on their enhanced structural integrity and more diverse ECM composition. Furthermore, the concentration of 30 mg/mL was chosen based on its minimal swelling, high level of porosity, and mechanical stability.

**FIG. 6.**
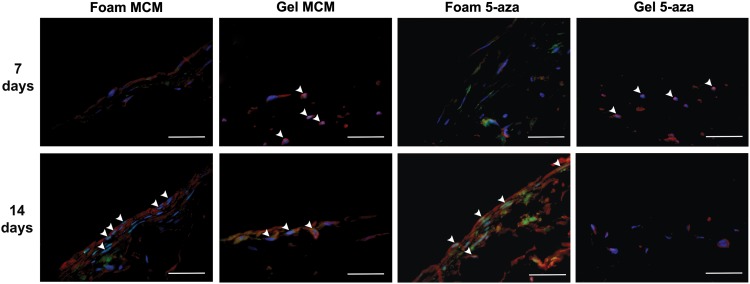
Representative IHC staining for Nkx2.5 (green) and Mlc2v (red) with DAPI counterstaining (blue) in the modified cardiomyogenic medium (MCM) and 5-azacytidine (5-aza)-stimulated pericardial fat adipose-derived stem/stromal cells (pfASCs) cultured on the DLV foams and collagen I gels for 7 or 14 days. White arrowheads indicate Nkx2.5 nuclear localization, indicative of transcription factor activation. In the DLV foams, Mlc2v expression was qualitatively enhanced at 14 days with both induction mediums, while in the collagen I gels, notable expression was only observed in the MCM group. Scale bars represent 200 μm.

**FIG. 7.**
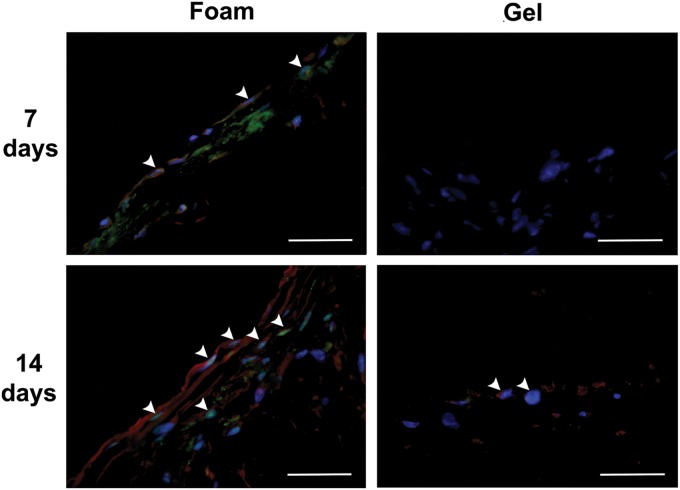
Representative IHC staining for Nkx2.5 (green) and Mlc2v (red) with DAPI counterstaining (blue) in the control group pfASCs cultured on the DLV foams and collagen I gels in complete medium for 7 or 14 days, suggesting an inductive effect of the cardiac extracellular matrix-derived foams on pfASC cardiomyogenic marker expression. White arrowheads indicate Nkx2.5 nuclear localization. Scale bars represent 200 μm.

Culturing the pfASCs on the foams induced with MCM or 5-aza stimulation resulted in high levels of Mlc2v expression at 14 days, along with nuclear localization of the cardiac transcription factor Nkx2.5 (Fig. 6 and Supplementary Fig. S3). Interestingly, the noninduced DLV foam controls maintained in complete medium demonstrated nuclear localization of Nkx2.5 at 7 and 14 days postinduction and enhanced Mlc2v expression at 14 days (Fig. 7 and Supplementary Fig. S4). In the collagen I gel groups, while nuclear Nkx2.5 was observed in the gels cultured in MCM at both 7 and 14 days, the Mlc2v expression levels were qualitatively lower than in the DLV foams. Furthermore, in the gels stimulated with 5-aza, Nkx2.5 was detected in the nuclei only at day 7 and limited Mlc2v expression was observed at both time points (Fig. 6 and Supplementary Fig. S5). In the noninduced collagen I gel controls, low levels of nuclear staining for Nkx2.5 were observed in a small number of cells at 14 days and there was very limited expression of Mlc2v at both time points (Fig. 7 and Supplementary Fig. S6). The gelatin-coated cover-slips showed low levels of Mlc2v expression at 14 days in the induced groups, but no nuclear localization of Nkx2.5 was observed under any conditions (Supplementary Fig. S7).

### Cardiac gene expression in pfASCs cultured on DLV foams and collagen I gels

**FIG. 8.**
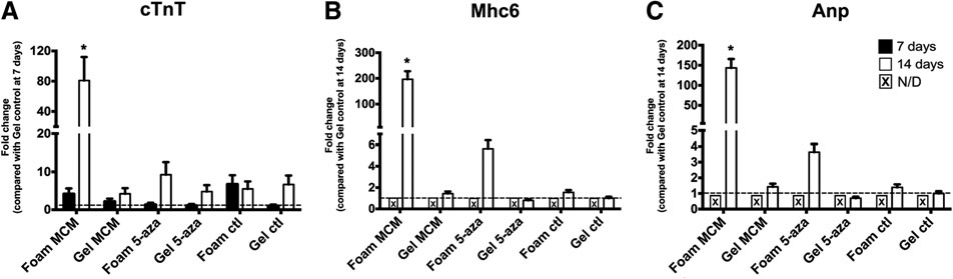
Gene expression analysis of the cardiomyogenic markers, cardiac troponin T type II (*cTnT*), myosin heavy chain 6 (*Mhc6*), and atrial natriuretic peptide (*Anp*), in pfASCs cultured on the DLV foams and collagen I gels at 7 and 14 days by real-time reverse transcriptase-polymerase chain reaction (RT-PCR) (*n* = 3). Gene expression was normalized to the geometric mean of the housekeeping genes (β-*actin* and tata box-binding protein [*TBP*]) and the comparative C_t_ method was employed using the collagen I gel controls at 7 (*cTnT*) or 14 (*Mhc6*, *Anp*) days as the calibrator. N/D, not detected. All data are expressed as mean ± SD. * Significantly different from all other groups (*p* < 0.05).

A follow-up gene expression study was conducted to further probe the influence of the cardiac ECM microenvironment on the early induction of downstream markers of pfASC cardiomyogenic differentiation. For the analysis of the cardiac sarcomere component *cTnT*, significantly higher expression levels were observed in the DLV foams cultured in MCM at 14 days compared with all other groups, with an 80.9 ± 31.0-fold increase relative to the noninduced collagen I gels at 7 days (Fig. 8A). In comparing the 5-aza-stimulated groups at 14 days, a trend for enhanced *cTnT* expression was observed in the DLV foams (9.2 ± 3.3-fold) relative to the collagen I gels (4.7 ± 1.7-fold). Under the conditions in the current study, no expression of the cardiac sarcomere component *Mhc6* or the secreted protein *Anp* was detected in any of the study groups at day 7 (Fig. 8B, C). At 14 days, the *Mhc6* and *Anp* expression levels were significantly enhanced in the DLV foams cultured in MCM compared with all other groups, with a 196.6 ± 31.4-fold and 143.5 ± 22.2-fold increase, respectively, relative to the noninduced collagen I gels. A trend for enhanced expression was also noted in the 5-aza-stimulated DLV foams, with a 5.6 ± 0.7-fold increase in *Mhc6* and 3.6 ± 0.5-fold increase in *Anp* relative to the noninduced collagen I gels. While not statistically significant, *Mhc6* and *Anp* expression was slightly elevated in the noninduced DLV foams, with a 1.5 ± 0.2-fold and 1.3 ± 0.1-fold increase, respectively, relative to the noninduced collagen I gels.

## Discussion

In recent years, efforts have been made to develop 3D ECM-derived bioscaffolds as alternatives to 2D culture systems for cardiac cell culture applications. In the present study, we fabricated and characterized novel, nonchemically cross-linked porous foams comprised entirely of decellularized porcine left ventricular myocardium. While chemical cross-linking is commonly employed to increase the mechanical stability of collagen scaffolds,^34–36^ it is known that it can impact scaffold properties, including degradation^37^ and stiffness,^20,38^ which are key mediators of cell behavior. Furthermore, most chemical cross-linkers are associated with cytotoxicity risks^35,36^ and may alter the bioactivity of the natural ECM.^39^ To circumvent the need for chemical cross-linking, we utilized the digestive enzyme, α-amylase, in place of pepsin, which is commonly employed in the generation of collagen foams^15^ and hydrogels.^18,20^ Unlike the proteolytic enzyme pepsin, α-amylase is postulated to increase collagen solubility in acetic acid by cleaving carbohydrate groups rather than peptide bonds.^21,40^ While pepsin digestion results in collagen fragments that produce mechanically weak gels, α-amylase treatment enables the production of robust scaffolds through controlled freezing and lyophilization that are stabilized through physical cross-linking of the collagen fibers.

To assess the flexibility of our new cell culture platform, we characterized the influence of the ECM preprocessing methods and concentration on a range of scaffold properties, including stiffness, porosity, and degradation. This comparison revealed differences in the structure, composition, and mechanical properties between the minced and cryomilled DLV foams, as well as in long-term stability in culture. In particular, the minced foams were shown to have a more structurally robust and compositionally heterogeneous ECM network, with conserved laminin expression. In the context of cardiac cell culture, the presence of laminin and fibronectin may be advantageous as these proteins have been shown to promote cell attachment and proliferation,^41,42^ as well as the cardiomyogenic differentiation of human ASCs^43^ and embryonic stem cells (ESCs).^44^

In terms of mechanical properties, our fabrication approach yielded DLV foams with a maximum Young's modulus of 5.57 ± 0.06 kPa for the 40 mg/mL minced foam group, which is ∼10-fold lower than what was reported by Rajabi-Zeleti et al. for non cross-linked pericardium foams.^15^ The higher stiffness displayed by the pericardium foams could be a consequence of differences in the structure and composition of the ECM in the source tissues as the pericardium has been shown to contain a higher fraction of fibrous collagen compared with the myocardium.^45,46^ In the context of cardiomyogenesis, the stiffness of our minced DLV foams may be favorable for induction along this lineage as it has been reported that ESC cardiac differentiation is enhanced when the cells are cultured on materials with a Young's modulus between 5 and 10 kPa.^47,48^ While previous studies with freeze-dried collagen scaffolds have demonstrated a direct relationship between collagen concentration and Young's modulus,^38,49^ our data indicate that the ECM preprocessing methods can also mediate the mechanical properties as the Young's modulus remained consistently low in the cryomilled DLV foams and was independent of the DLV concentration and scaffold porosity.

As proof of concept to test the potential of the DLV foams as a 3D culture support for cardiac studies, we investigated the early cardiomyogenic differentiation of pfASCs on the foams and collagen I gel controls. In addition to the established MCM, we chose to investigate the DNA demethylating agent, 5-aza, as it has been one of the most commonly explored cardiomyogenic factors for MSC differentiation, although it has yielded controversial results in terms of its efficacy with ASCs.^25,50,51^ Interestingly, in differentiation studies on bone marrow-derived MSCs and ASCs, preincubation with 5-aza has also been shown to have nonspecific inductive effects, increasing not only cardiomyogenic differentiation^52^ but also chondrogenesis^53^ and osteogenesis^54^ in their respective inductive media. These data suggest that 5-aza may nonspecifically alter the expression of certain genes associated with differentiation, enhancing the sensitivity of the stem cells to a range of inductive factors, rather than by directly driving differentiation toward a specific lineage.

Our results indicate that pfASC cardiomyogenesis was enhanced in the DLV foams relative to the collagen I gels, with MCM having a more potent inductive effect on the expression of cardiomyocyte gene markers compared with 5-aza stimulation. Furthermore, in comparing the 5-aza-treated pfASCs seeded on the DLV foams and purified collagen I gels, notable expression of cardiomyogenic protein markers was only observed in the DLV foam group. Taken together, these results suggest that the cardiac ECM-derived DLV foams may provide a more permissive environment for the induction of pfASC cardiomyogenesis. Supporting our findings, a synergistic effect between 5-aza stimulation and the cardiac ECM components, laminin and fibronectin, on ASC cardiomyogenic marker expression has been previously reported in 2D culture on TCPS^43^ and in 3D culture on poly-(lactic-*co*-glycolic) acid microspheres.^55^ Moreover, several studies have demonstrated that cardiomyogenesis was promoted by culturing cardiac progenitor cells (CPCs) and ESCs on cardiac ECM-derived hydrogels.^18,20,56^ Our data further support that the ECM composition may play an important role in orchestrating the initiation and progression of differentiation.^15,18,56,57^

On a molecular level, one of the pivotal mechanisms involved in the progression of cardiomyogenesis is the nuclear translocation of Nkx2.5,^58^ which activates the transcription of cardiac-specific genes, including *Mlc2v*, *Mhc6*, and *Anp*.^58–61^ Our results are consistent with the early stages of this process, with Nkx2.5 nuclear localization observed at 14 days in the induced DLV foam groups, along with increased expression of downstream cardiac markers at both the gene and protein levels. In addition to ECM composition, it is important to recognize that other elements may be influencing cell differentiation, including substrate topography,^62,63^ the distribution pattern of bioactive cues,^64^ the stiffness of the scaffold,^47,48^ and the level of cell–cell contact.^25,65^ As previously discussed, while the ECM composition may have been a key mediator of the enhanced response in the foams, the biomechanical properties may also have been favorable for induction along this lineage. In this context, it has been postulated that recapitulating the biomechanical environment of the developing heart^66,67^ may enhance regenerative and proliferative cell behaviors,^57^ thereby supporting the rationale for the use of lower stiffness culture substrates.

Interestingly, pfASCs cultured on the DLV foams in control medium conditions also displayed nuclear localization of Nkx2.5 and Mlc2v protein expression. In terms of scaffold-induced cardiomyogenic differentiation, Rajabi-Zeleti et al. have previously reported significantly higher expression of cardiac transcription factors and other cardiac markers at the gene level in CPCs cultured in control medium on pericardium foams compared with collagen I gels and decellularized pericardium.^15^ One possible explanation for the improved differentiation on the more highly processed scaffolds could lie in the enhanced infiltration of the cells into the foams due to their higher porosity relative to the intact decellularized tissue.^15,68^ While our results suggest that the DLV foams had an inductive effect on pfASC cardiomyogenesis, the expression of cardiac gene markers was generally low in this treatment group, suggesting the cells had a more immature phenotype in the absence of an inductive medium.

## Conclusion

In conclusion, we have successfully established straightforward methods to fabricate 3D porous foams derived entirely from decellularized left ventricular myocardium, which are stable in culture without the need for chemical cross-linking. The scaffolds are easy to handle with forceps and can be synthesized in a broad range of sizes and geometries. Through detailed characterization studies, we have demonstrated that the foam properties can be tuned to some extent by varying the DLVsus concentration. Furthermore, we have established that physically processing the ECM through mincing versus cryomilling impacts the structure, composition, and mechanical properties of the resultant foams. Our data suggest that the DLV foams may represent a useful platform for cardiac cell culture studies. Based on our preliminary findings that the DLV foams provide a supportive microenvironment for inducing human pfASC cardiomyogenesis, our ECM-derived scaffolds may be a useful substrate for promoting the lineage-specific differentiation of other stem and progenitor cell sources. In future studies, it would also be interesting to explore the response of primary cardiomyocytes within the foams as a step toward establishing more complex cardiac cell culture systems that better recapitulate the native cellular milieu.

## Supplementary Material

Supplemental data

Supplemental data

Supplemental data

Supplemental data

Supplemental data

Supplemental data

Supplemental data

## Abbreviations Used

5-aza: 5-azacytidine
Anp: atrial natriuretic peptide
Coll I: collagen I
Coll IV: collagen IV
CPC: cardiac progenitor cell
cTnT: cardiac troponin T type II
DLV: decellularized porcine left ventricle
DLVsus: DLV suspension
DMEM-lg: low-glucose Dulbecco's modified Eagle's medium
ECM: extracellular matrix
EWC: equilibrium water content
FE: finite element
FN: fibronectin
IHC: immunohistochemical
LN: laminin
MCM: modified cardiomyogenic medium
Mhc6: myosin heavy chain 6
Mlc2v: ventricular myosin light chain 2
PBS: phosphate-buffered saline
pfASC: pericardial fat adipose-derived stem cell
PMSF: phenylmethanesulfonyl fluoride
SD: standard deviation
SEM: scanning electron microscopy
SR: swelling ratio
TBP: tata box-binding protein
TCPS: tissue culture polystyrene
TE: Tris-EDTA
TU: Tris-urea
