# Supplementary material for: Porous, Ventricular Extracellular Matrix-Derived Foams as a Platform for Cardiac Cell Culture
Source: Biores Open Access. 2015 Oct 1;4(1):374–88. doi: 10.1089/biores.2015.0030 (PMC4598938; doi:10.1089/biores.2015.0030)
Supplement: Supplemental data [file Supp_Fig1.pdf]

Supplementary Data

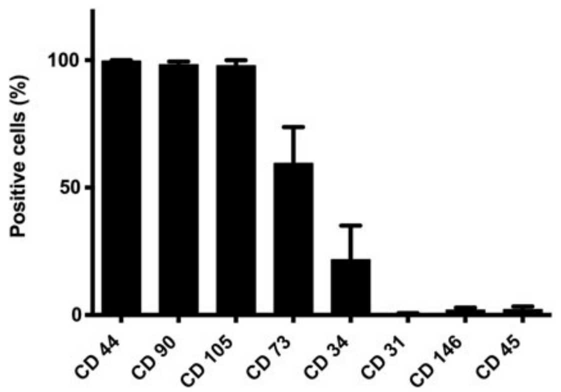

SUPPLEMENTARY FIG. S1. Immunophenotype analysis of passage 4 pericardial fat adipose-derived stem/stromal cells (pfASCs). All data are expressed as mean ± standard deviation ($n = 3$, $N = 3$).
