# Supplementary material for: Porous, Ventricular Extracellular Matrix-Derived Foams as a Platform for Cardiac Cell Culture
Source: Biores Open Access. 2015 Oct 1;4(1):374–88. doi: 10.1089/biores.2015.0030 (PMC4598938; doi:10.1089/biores.2015.0030)
Supplement: Supplemental data [file Supp_Fig2.pdf]

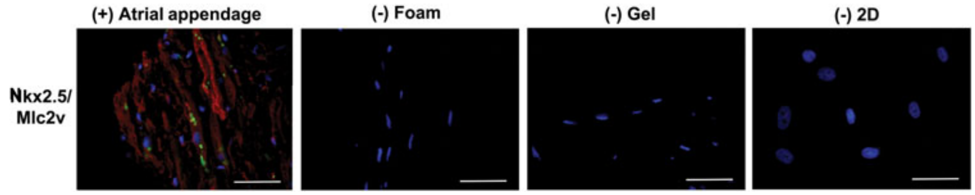

SUPPLEMENTARY FIG. S2. Representative images of Nkx2.5 (green), Mlc2v (red) and DAPI (blue) staining in the positive (+) and negative (–) controls included in the Nkx2.5/Mlc2v immunohistochemical (IHC) costaining analysis. Human atrial appendage was used as a tissue positive control. The negative controls (no primary antibody) show representative images of the pfASCs cultured on the various substrates (decellularized porcine left ventricle [DLV] foams, collagen I gels, and 2D gelatin-coated glass cover-slips) in modified cardiomyogenic medium (MCM) for 14 days. Overall, the controls confirmed that the antibody labeling was specific. Scale bars represent 200 μm.
