# Supplementary material for: Porous, Ventricular Extracellular Matrix-Derived Foams as a Platform for Cardiac Cell Culture
Source: Biores Open Access. 2015 Oct 1;4(1):374–88. doi: 10.1089/biores.2015.0030 (PMC4598938; doi:10.1089/biores.2015.0030)
Supplement: Supplemental data [file Supp_Fig3.pdf]

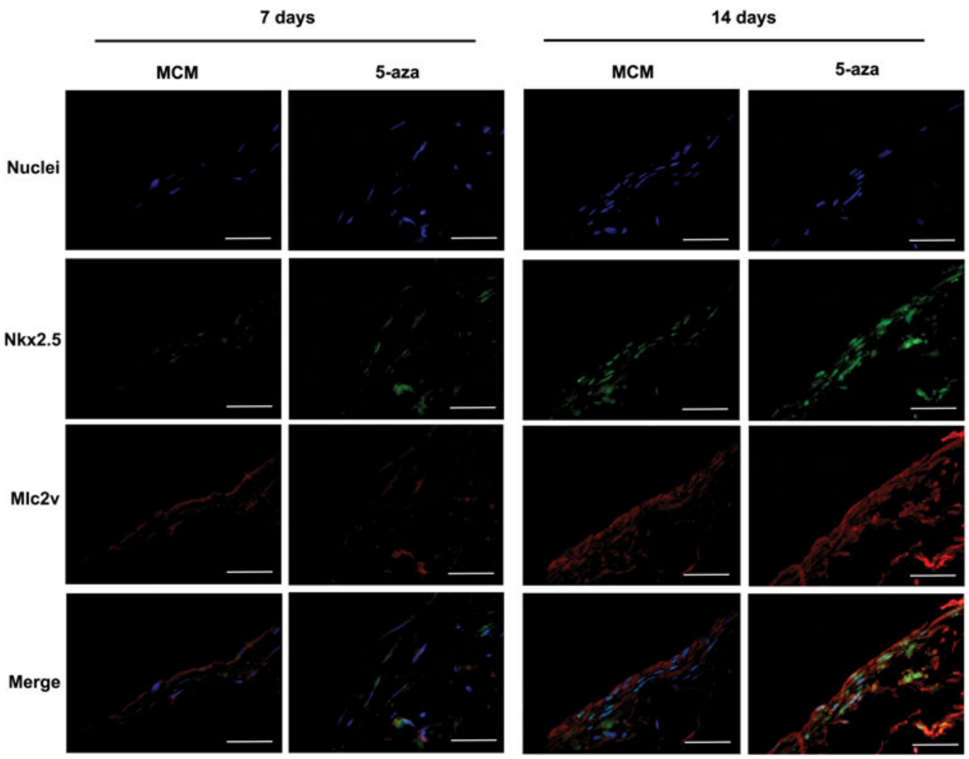

SUPPLEMENTARY FIG. S3. Representative images of the individual and merged channels for the IHC staining of DAPI (blue), Nkx2.5 (green) and Mlc2v (red) in the MCM and 5-azacytidine (5-aza)-treated DLV foams. Scale bars represent 200 μm.
