# Supplementary material for: Porous, Ventricular Extracellular Matrix-Derived Foams as a Platform for Cardiac Cell Culture
Source: Biores Open Access. 2015 Oct 1;4(1):374–88. doi: 10.1089/biores.2015.0030 (PMC4598938; doi:10.1089/biores.2015.0030)
Supplement: Supplemental data [file Supp_Fig4.pdf]

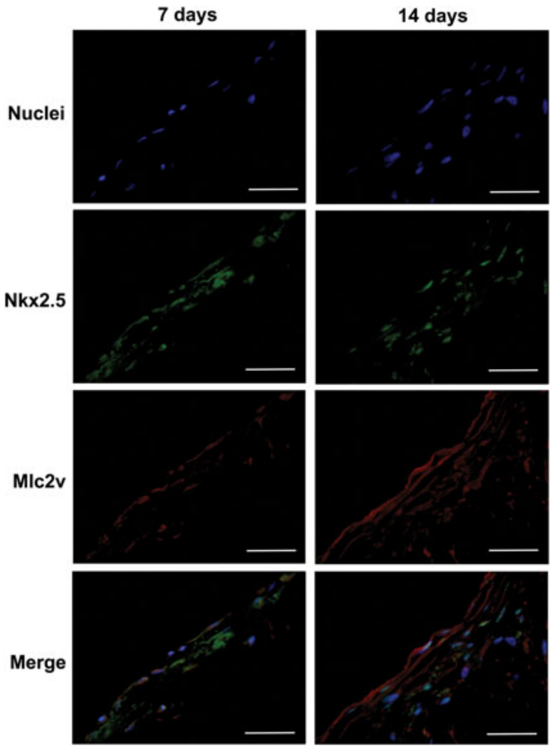

SUPPLEMENTARY FIG. S4. Representative images of the individual and merged channels for the IHC staining of DAPI (blue), Nkx2.5 (green), and Mlc2v (red) in the DLV foam control group maintained in complete medium. Scale bars represent 200 μm.
