# Supplementary material for: Porous, Ventricular Extracellular Matrix-Derived Foams as a Platform for Cardiac Cell Culture
Source: Biores Open Access. 2015 Oct 1;4(1):374–88. doi: 10.1089/biores.2015.0030 (PMC4598938; doi:10.1089/biores.2015.0030)
Supplement: Supplemental data [file Supp_Fig6.pdf]

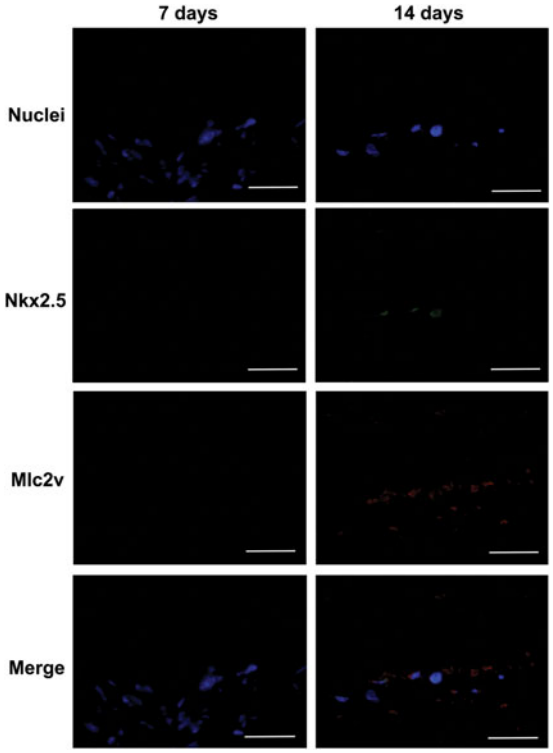

SUPPLEMENTARY FIG. S6. Representative images of the individual and merged channels for the IHC staining of DAPI (blue), Nkx2.5 (green), and Mlc2v (red) in the collagen I gel control group maintained in complete medium. Scale bars represent 200 μm.
