# Supplementary material for: Porous, Ventricular Extracellular Matrix-Derived Foams as a Platform for Cardiac Cell Culture
Source: Biores Open Access. 2015 Oct 1;4(1):374–88. doi: 10.1089/biores.2015.0030 (PMC4598938; doi:10.1089/biores.2015.0030)
Supplement: Supplemental data [file Supp_Fig7.pdf]

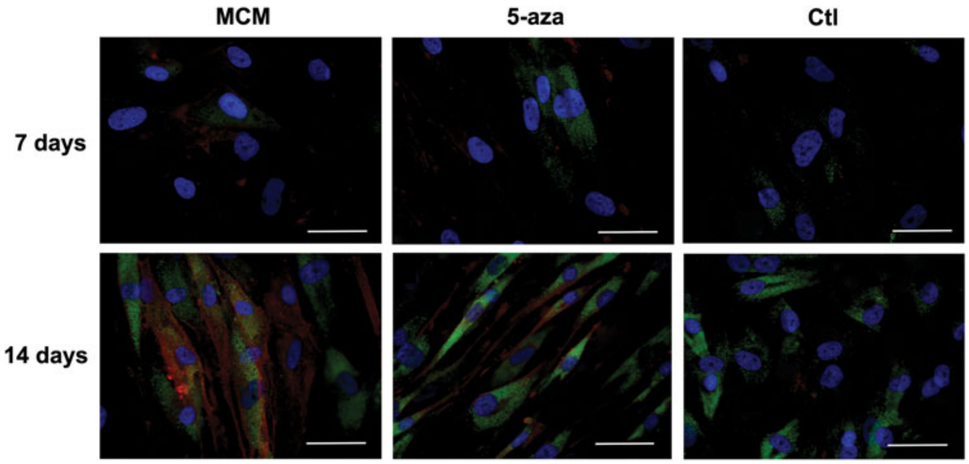

SUPPLEMENTARY FIG. S7. Representative immunocytochemical staining for DAPI (blue), Nkx2.5 (green), and Mlc2v (red) in the pfASCs cultured on gelatin-coated glass cover-slips. Scale bars represent 200 μm.
